# Design of a Measurement System for Simultaneously Measuring Six-Degree-Of-Freedom Geometric Errors of a Long Linear Stage

**DOI:** 10.3390/s18113875

**Published:** 2018-11-10

**Authors:** Chien-Sheng Liu, Yu-Fan Pu, Yu-Ta Chen, Yong-Tai Luo

**Affiliations:** 1 Department of Mechanical Engineering, National Cheng Kung University, Tainan City 70101, Taiwan; 2 Department of Mechanical Engineering, National Chung Cheng University, Chiayi County 62102, Taiwan; pupufan@hotmail.com.tw (Y.-F.P.); michael102518@gmail.com (Y.-T.C.); aa0983426959@gmail.com (Y.-T.L.); 3 Advanced Institute of Manufacturing with High-tech Innovations, National Chung Cheng University, Chiayi County 62102, Taiwan

**Keywords:** geometric errors, linear stage, error measurement, error analysis, machine tool, multiple-degree-of-freedom error

## Abstract

This study designs and characterizes a novel precise measurement system for simultaneously measuring six-degree-of-freedom geometric motion errors of a long linear stage of a machine tool. The proposed measurement system is based on a method combined with the geometrical optics method and laser interferometer method. In contrast to conventional laser interferometers using only the interferometer method, the proposed measurement system can simultaneously measure six-degree-of-freedom geometric motion errors of a long linear stage with lower cost and faster operational time. The proposed measurement system is characterized numerically using commercial software ZEMAX and mathematical modeling established by using a skew-ray tracing method, a homogeneous transformation matrix, and a first-order Taylor series expansion. The proposed measurement system is then verified experimentally using a laboratory-built prototype. The experimental results show that, compared to conventional laser interferometers, the proposed measurement system better achieves the ability to simultaneously measure six-degree-of-freedom geometric errors of a long linear stage (a traveling range of 250 mm).

## 1. Introduction

Recently, in accordance with the increasing market demand for ultraprecise machine tools to machine complicated workpiece surfaces, multi-axis machine tools have become more and more important and play a crucial role in the manufacturing and machining field [1,2]. In order to produce multi-axis machine tools with high accuracy and repeatability, inspection and compensation techniques for static and dynamic errors of machine tools are necessary [3,4,5]. At present, for multi-axis machine tools, linear precision stages are the key components, and have been widely used as the basis for linear motion, and constrain its motion to a desired direction or posture [6,7]. However, due to the deviations caused by manufacturing imperfections, assembly, misalignments, structural deflections and so on, a linear precision stage will inherently have six-degree-of-freedom (6DOF) geometric motion errors, including three linear errors (positioning error *δ_x_*, horizontal straightness error *δ_y_*, and vertical straightness error *δ_z_*) and three angular errors (pitch error *ε_y_*, yaw error *ε_z_*, and roll error *ε_x_*) [8,9]. As a result, in order to improve the accuracy and repeatability of the multi-axis machine tools, 6DOF geometric motion errors of the linear precision stage should be accurately identified and effectively compensated [10,11]. 

Traditionally, the laser interferometer, the well-known non-contact measuring instrument with high resolution and long range, has been widely implemented to measure geometric motion errors of the linear precision stage for multi-axis machine tools. However, it can only measure a single geometric motion error of the linear precision stage in each experimental setup. Therefore, it is a time-consuming work to completely measure all geometric motion errors of the linear precision stage using the laser interferometer [7,11,12]. In recent years, some measurement systems for simultaneously measuring multi-DOF geometric motion errors of a long linear stage of a machine tool have been developed in the literature [13,14,15,16,17,18,19,20,21,22,23,24,25,26,27,28,29,30,31]. For example, for contact measurement, Mura proposed a novel measurement device, consisting of six displacement sensors mounted as a parallel mechanism based on the Stewart theory. By applying the direct kinematic equations to convert the six displacements read from the six displacement sensors into the three translations and rotations, it can measure the global deformation of a component [14,15,16]. However, it is difficult to measure the positioning error *δ_x_* for a long traveling range (>200 mm) with high accuracy and repeatability due to its inherent limits. 

Among those measurement systems, non-interferometric optical sensing technologies have been verified to be superior to others in many aspects, including compactness, immunity to electromagnetic interference, and noncontact measurement [7,10]. For example, Lee et al. presented a 6DOF geometric error measurement system that can be applied to the simultaneous measurement of six geometric error components of the moving axes of a meso-scale machine tool. The measured traveling range is 4 mm. The presented measurement system consists of a laser module constructed by a laser diode, a cube beam splitter (BS) and three two-dimensional position sensitive detectors (PSDs), and an additional cube beam splitter (BS) [10]. Kuang et al. proposed a novel and single four-degree-of-freedom (4DOF) laser measuring system in which only a cube corner retro-reflector and a BS were adopted to sense the straightness errors and angular errors, respectively [20]. However, it is also difficult to measure the positioning error *δ_x_* for a long traveling range (> 200 mm) with high accuracy and repeatability due to its inherent limits. Therefore, Fang et al. proposed a measurement system to simultaneously measure 6DOF geometric errors. The measurement method is based on a combination of laser interferometry and laser fiber collimation. Positioning error measurement was achieved by laser interferometry, and other five-degree-of-freedom (5DOF) geometric motion errors were obtained by fiber collimation measurement [28,29,30,31]. However, to the best of the current authors’ knowledge, these techniques are very few.

As a result, in this study, a novel precise and simple measurement system for simultaneously measuring 6DOF geometric motion errors of a long linear stage has been proposed to provide another solution. The proposed measurement system is based on a method combined with the geometrical optics method (non-interferometric optical sensing technologies) and laser interferometer method. In the proposed approach, a commercial laser interferometer was combined into the proposed measurement system. The structure and the principles of the proposed measurement system are described in detail as follows. The proposed measurement system is characterized numerically, and then verified experimentally using a laboratory-built prototype. Finally, some brief concluding remarks are presented.

## 2. Structure Layout and Measuring Principle

**Figure 1:**
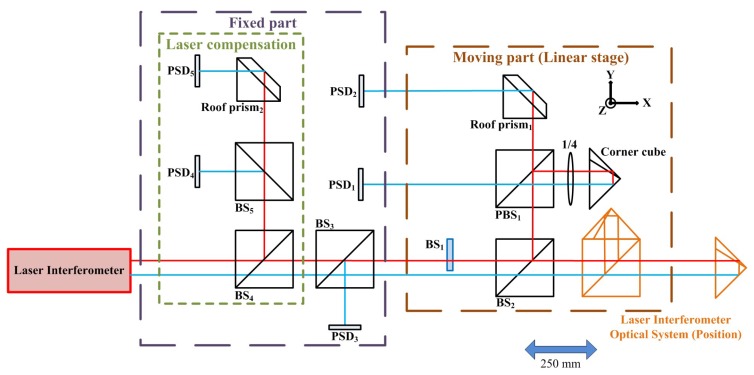
Structure of proposed measurement system.

The structure layout of the proposed measurement system for simultaneously measuring 6DOF geometric motion errors of a long linear stage is shown in Figure 1. The proposed measurement system consists of two parts, namely a moving part attached on the measured long linear stage and a fixed part. The proposed measurement system is constituted of a laser interferometer, five two-dimensional PSDs, five BSs, two roof prisms, and one corner cube. Among them, the PSD utilizes a silicon photodiode-based pincushion tetra lateral sensor (Newport, CA, USA, CONEX-PSD9, position sensitivity of 0.5 μm) to accurately measure the displacement of an incident beam relative to the calibrated center. Unlike quadrant detectors, the improved tetra-lateral effect diode is highly linear over the full sensor size. In the proposed approach, the commercial laser interferometer is combined into the proposed measurement system to measure the positioning error of the linear stage.

**Figure 2:**
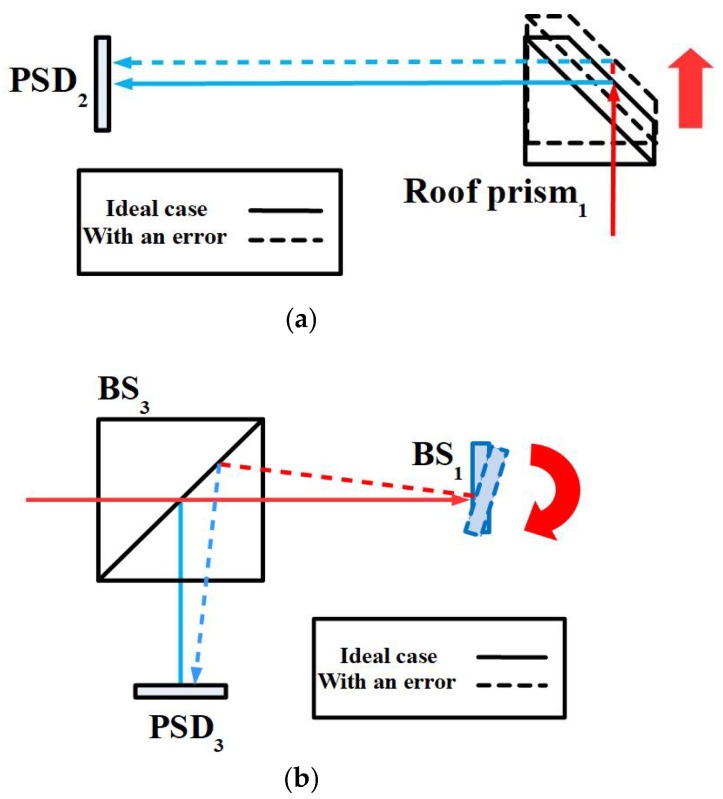

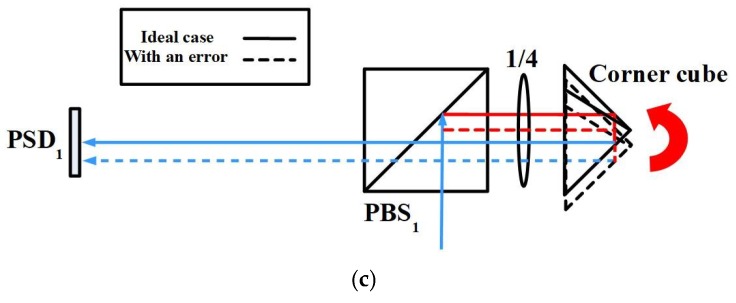
Motion error-induced changes in positions of light spots on position sensitive detectors (PSDs). (**a**) Linear error along the Y-axis (*δ_y_*) or Z-axis (*δ_z_*). (**b**) Angular error along the Z-axis (*ε_z_*) or Y-axis (*ε_y_*). (**c**) Angular error along the X-axis (*δ_x_*).

When the linear stage with 6DOF geometric errors moves to different positions, the optical paths of the laser beams are changed in the proposed measurement system and then the positions of the light spots on the PSDs are also changed. Figure 2 illustrates the motion error-induced changes in the positions of the light spots on the PSDs for the horizontal straightness error *δ_y_*, vertical straightness error *δ_z_*, pitch error *ε_y_*, yaw error *ε_z_*, and roll error *ε_x_*, respectively.

**Figure 3:**
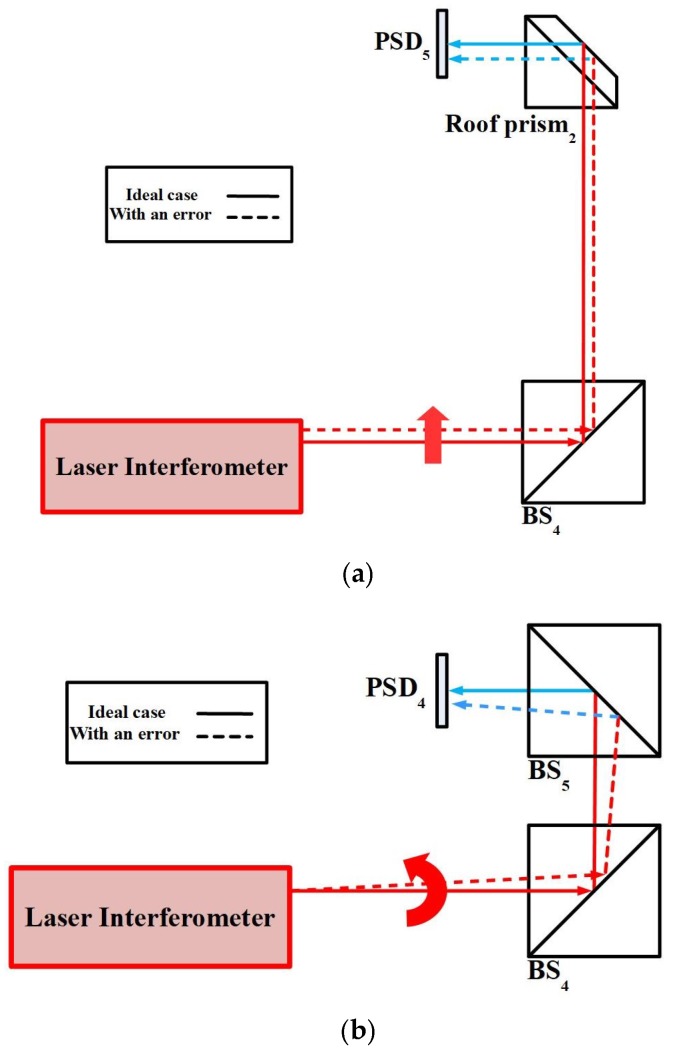
Fluctuation-induced changes in positions of light spots on PSDs. (**a**) Linear fluctuation along the Y-axis or Z-axis. (**b**) Angular fluctuation along the Y-axis or Z-axis.

However, the timing fluctuations of the laser source introduce noise, thereby degrading the measurement accuracy [32,33,34,35]. As a result, this study proposes a method to measure and compensate for fluctuations in the laser beam geometry. As shown in Figure 3, two PSDs (PSD_4_ and PSD_5_) are used to measure linear fluctuations (*δ_ly_* and *δ_lz_*) and angular fluctuations (*ε_ly_* and *ε_lz_*) of the laser source in this study. As stated in Section 3, a forward light ray tracing method is used to follow the laser beam. From these data, the effects of 6DOF geometric errors of the linear stage on the light spot positions on the PSDs are determined, and a reverse derivation is applied to find the 6DOF geometric errors of the linear stage from the light spot position information [7]. Subsequently the measurement accuracy of the proposed measurement system can be improved by compensating the measured linear and angular fluctuations of the laser source. As a result, the 6DOF geometric errors of the linear stage can be obtained by analyzing the position information of the light spots on the PSDs and the optical paths in the proposed measurement system.

## 3. Numerical Simulation and Mathematical Model

**Figure 4:**
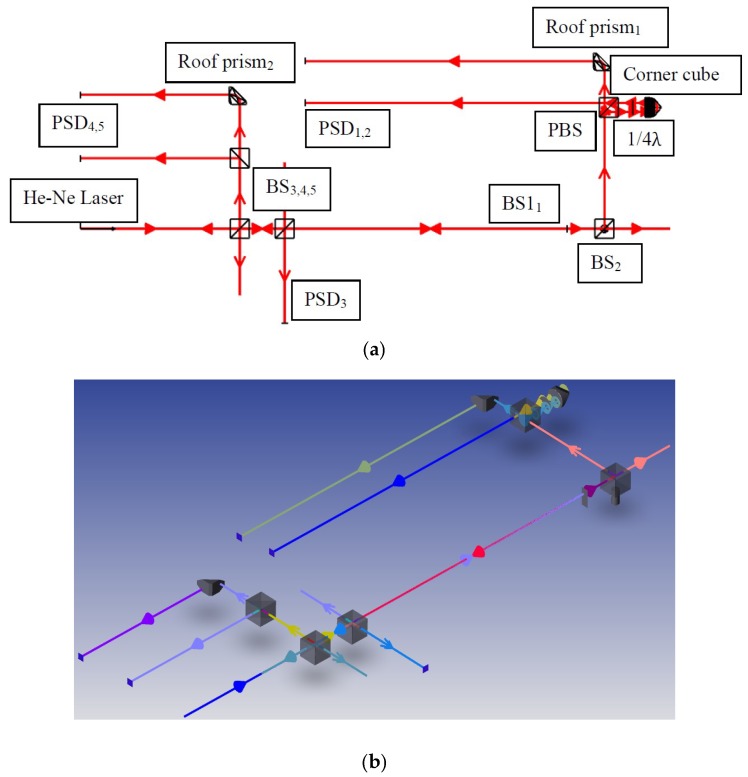
ZEMAX. (**a**) 2D and (**b**) 3D optical models of proposed measurement system.

**Figure 5:**
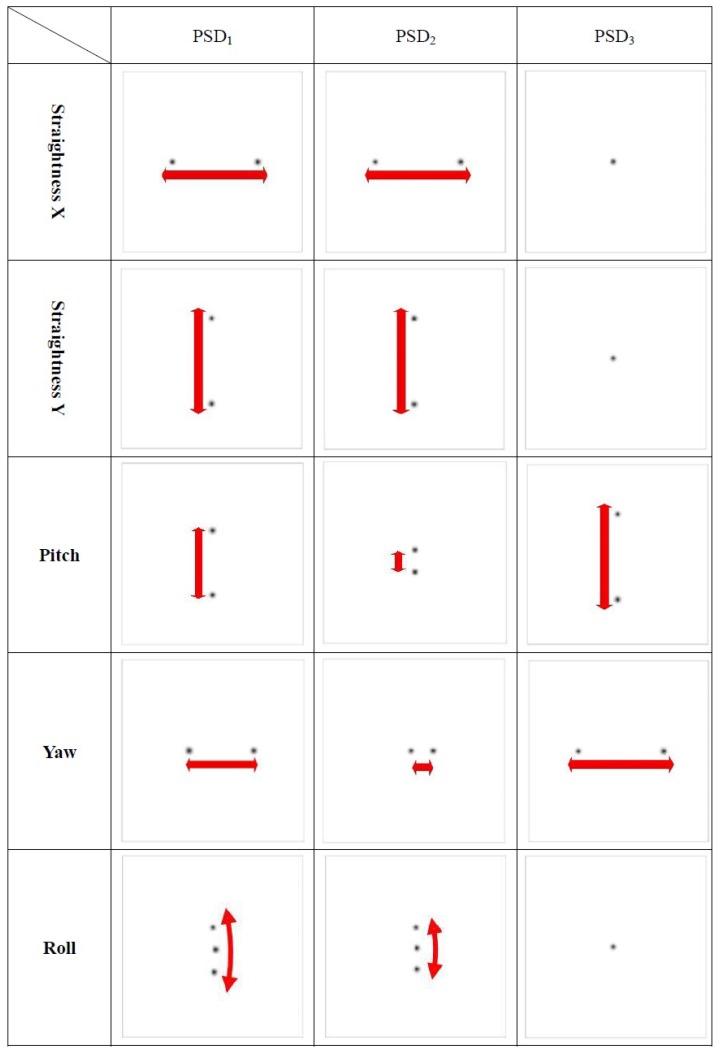
Simulation results for variation of positions of light spots on PSDs with motion errors.

In this study, the ray trace function of ZEMAX software was used to verify the measuring performance of the proposed measurement system and simulate the positions of the light spots on the PSDs with qualitative analysis. Figure 4 shows the ZEMAX optical models of the proposed measurement system. Figure 5 illustrates the optical simulation results obtained for the changes in the laser spots on the PSDs with different horizontal straightness errors, vertical straightness errors, pitch errors, yaw errors, and roll errors, respectively. It is noted that the numerical simulation of the positioning error is omitted, because the measurement of the positioning error is carried out using the commercial laser interferometer. These optical simulation results imply the feasibility of the proposed measurement system.

**Figure 6:**
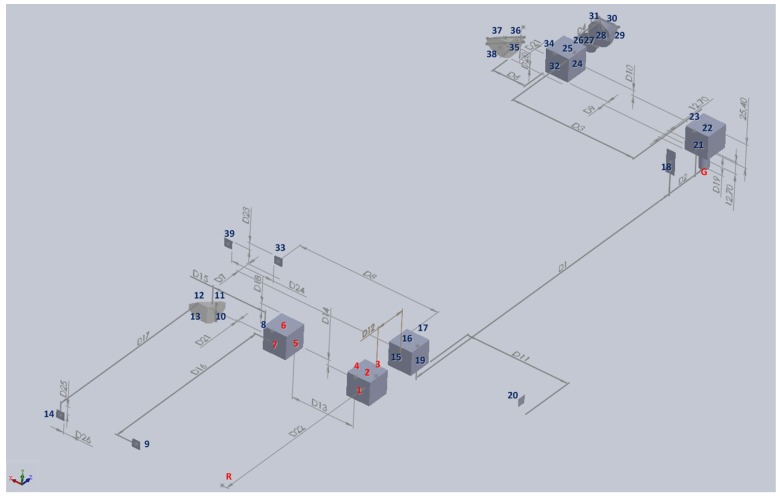
39 boundary surfaces of proposed measurement system.

Since a detailed mathematical derivation of the proposed measurement system could be found in our previous publication [7], this subsection only briefly reviews it and presents the difference. To calculate the relation between the individual 6DOF geometric errors and fluctuations of the laser source and the position information of the light spots with quantitative analysis, a skew-ray tracing method and a homogeneous transformation matrix (HTM) are adopted here. By using the HTM, we can define the coordinate frame of each optical boundary relative to a reference coordinate system, as shown in Figure 6, and establish the light beam tracing equations. 

Following the algorithm of the flat-boundary skew-ray tracing method which was shown in our previous publication [35,36,37,38,39], ${}^{R}A_{i}$ denotes the transfer matrix from each optical device (*i*) coordinate system to the reference coordinate system (*R*), and is shown as follows:

(1) $${}^{R}A_{i}=\left[\begin{array}{cccc} I_{ix} & J_{ix} & K_{ix} & t_{ix}\\ I_{iy} & J_{iy} & K_{iy} & t_{iy}\\ I_{iz} & J_{iz} & K_{iz} & t_{iz}\\ 0 & 0 & 0 & 1 \end{array}\right]\;$$

**Figure 7:**
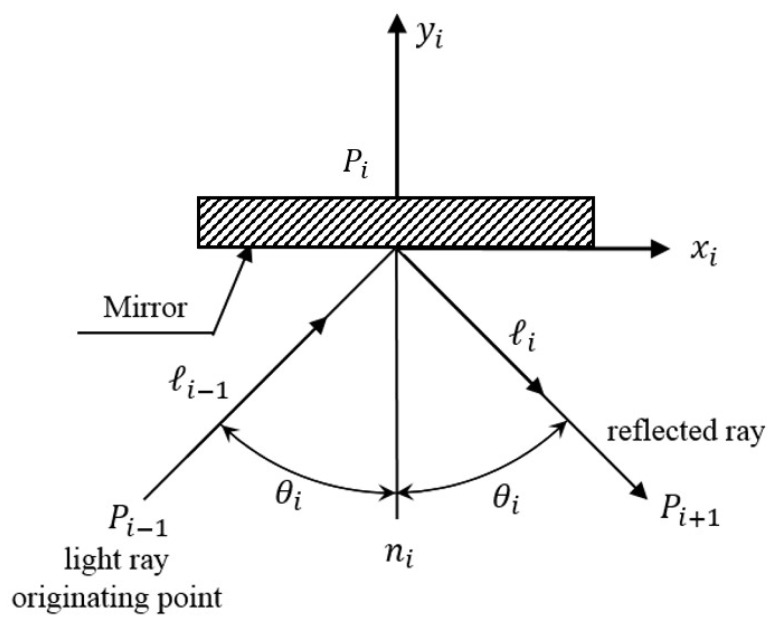
Laser ray incident upon a flat surface.

As shown in Figure 7, a laser beam originates from $P_{i-1}={\left[\begin{array}{cccc} P_{i-1x} & P_{i-1y} & P_{i-1z} & 1 \end{array}\right]}^{T}$ and is incident on a flat surface along the unit directional vector ${ℐ}_{i-1}={\left[\begin{array}{cccc} {ℐ}_{i-1x} & {ℐ}_{i-1y} & {ℐ}_{i-1z} & 0 \end{array}\right]}^{T}$. When the ray impacts the flat surface, if *λ_i_* is the vector from the source *P_i − 1_* to the destination point *P_i_*, then *λ_i_* is as follows:

(2) $$P_{i}={\left[\begin{array}{cccc} P_{ix} & P_{iy} & P_{iz} & 1 \end{array}\right]}^{T}={\left[\begin{array}{cccc} P_{i-1x}+{ℐ}_{i-1x}\lambda_{i} & P_{i-1y}+{ℐ}_{i-1y}\lambda_{i} & P_{i-1z}+{ℐ}_{i-1z}\lambda_{i} & 1 \end{array}\right]}^{T}\;$$

(3) $$\lambda_{i}=\frac{-(I_{iz}P_{i-1x}+J_{iz}P_{i-1y}+K_{iz}P_{i-1z}+t_{iz})}{I_{iz}{ℐ}_{i-1x}+J_{iz}{ℐ}_{i-1y}+K_{iz}{ℐ}_{i-1z}}=\frac{-B_{i}}{G_{i}}\;$$

According to Snell’s Law, the unit directional vector ${ℐ}_{i}$ of the reflected ray is as follows:

(4) $${ℐ}_{i}={\left[\begin{array}{cccc} {ℐ}_{ix} & {ℐ}_{iy} & {ℐ}_{iz} & 0 \end{array}\right]}^{T}={\left[\begin{array}{cccc} {ℐ}_{i-1x}-2I_{iz}G_{i} & {ℐ}_{i-1y}-2J_{iz}G_{i} & {ℐ}_{i-1z}-2K_{iz}G_{i} & 0 \end{array}\right]}^{T}\;$$

When the laser beam is incident upon the next flat surface, the previous point of incidence is the origin of the light source and the unit directional vector of the reflected ray is that of the incident ray. Tracing the laser ray path in the optical system chronologically, then the derivation of the forward ray tracing is used to locate the image centroid coordinates of the light spots on the five PSDs as follows:

*X*_PSD1_ = *F_X_*_1_(*δ_x_*, *δ_y_*, *δ_z_*, *ε_y_*, *ε_z_*, *ε_x_*, *δ_ly_*, *δ_lz_*, *ε_ly_*, *ε_lz_*), (5)

*Y*_PSD1_ = *F_Y_*_1_(*δ_x_*, *δ_y_*, *δ_z_*, *ε_y_*, *ε_z_*, *ε_x_*, *δ_ly_*, *δ_lz_*, *ε_ly_*, *ε_lz_*), (6)

*X*_PSD2_ = *F_X2_*(*δ_x_*, *δ_y_*, *δ_z_*, *ε_y_*, *ε_z_*, *ε_x_*, *δ_ly_*, *δ_lz_*, *ε_ly_*, *ε_lz_*), (7)

*Y*_PSD2_ = *F_Y2_*(*δ_x_*, *δ_y_*, *δ_z_*, *ε_y_*, *ε_z_*, *ε_x_*, *δ_ly_*, *δ_lz_*, *ε_ly_*, *ε_lz_*), (8)

*X*_PSD3_ = *F_X3_*(*δ_x_*, *δ_y_*, *δ_z_*, *ε_y_*, *ε_z_*, *ε_x_*, *δ_ly_*, *δ_lz_*, *ε_ly_*, *ε_lz_*), (9)

*Y*_PSD3_ = *F_Y3_*(*δ_x_*, *δ_y_*, *δ_z_*, *ε_y_*, *ε_z_*, *ε_x_*, *δ_ly_*, *δ_lz_*, *ε_ly_*, *ε_lz_*), (10)

*X*_PSD4_ = *F_X4_*(*δ_ly_*, *δ_lz_*, *ε_ly_*, *ε_lz_*), (11)

*Y*_PSD4_ = *F_Y4_*(*δ_ly_*, *δ_lz_*, *ε_ly_*, *ε_lz_*), (12)

*X*_PSD5_ = *F_X5_*(*δ_ly_*, *δ_lz_*, *ε_ly_*, *ε_lz_*), (13)

*Y*_PSD5_ = *F_Y5_*(*δ_ly_*, *δ_lz_*, *ε_ly_*, *ε_lz_*), (14)

where *X*_PSD*i*_ (*i* = 1, 2, 3, 4, and 5) and *Y*_PSD*i*_ (*i* = 1, 2, 3, 4, and 5) are the image centroid coordinates of the light spot on PSD*i* in the *X*-direction and *Y*-direction, respectively. 

Finally, by using a reverse mathematical derivation and a Taylor series expansion, we can simultaneously obtain the 6DOF geometric errors of the linear stage as follows:

*δ_y_* = *G_δy_* (*X*_PSD1_, *Y*_PSD1_, *X*_PSD2_, *Y*_PSD2_, *X*_PSD3_, *Y*_PSD3_, *X*_PSD4_, *Y*_PSD4_, *X*_PSD5_, *Y*_PSD5_), (15)

*δ_z_* = *G_δz_* (*X*_PSD1_, *Y*_PSD1_, *X*_PSD2_, *Y*_PSD2_, *X*_PSD3_, *Y*_PSD3_, *X*_PSD4_, *Y*_PSD4_, *X*_PSD5_, *Y*_PSD5_), (16)

*ε_y_* = *G_εy_* (*X*_PSD1_, *Y*_PSD1_, *X*_PSD2_, *Y*_PSD2_, *X*_PSD3_, *Y*_PSD3_, *X*_PSD4_, *Y*_PSD4_, *X*_PSD5_, *Y*_PSD5_), (17)

*ε_z_* = *G_εz_* (*X*_PSD1_, *Y*_PSD1_, *X*_PSD2_, *Y*_PSD2_, *X*_PSD3_, *Y*_PSD3_, *X*_PSD4_, *Y*_PSD4_, *X*_PSD5_, *Y*_PSD5_), (18)

*ε_x_* = *G_εx_* (*X*_PSD1_, *Y*_PSD1_, *X*_PSD2_, *Y*_PSD2_, *X*_PSD3_, *Y*_PSD3_, *X*_PSD4_, *Y*_PSD4_, *X*_PSD5_, *Y*_PSD5_), (19)

In this study, we only introduce the basic background of the HTM and the skew-ray tracing method to avoid repeat. For more comprehensive coverage, the reader is referred to [35,36,37,38,39] to avoid too much word repetition. 

## 4. Experimental characterization

**Figure 8:**
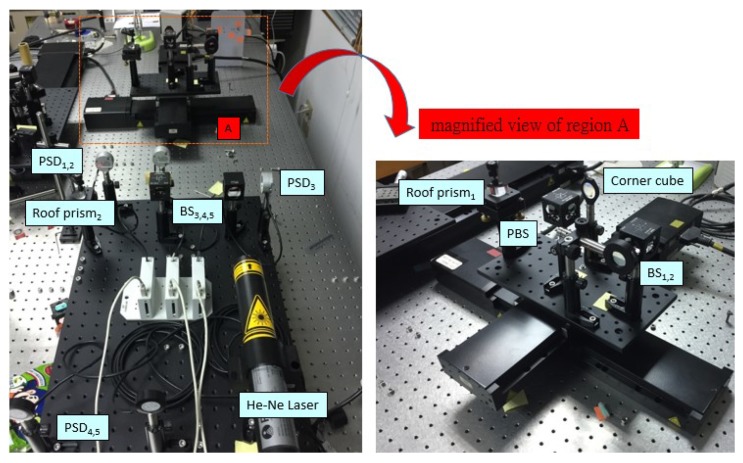
Photograph of laboratory-built prototype.

As shown in Figure 8, the validity of the proposed measurement system was verified by means of a laboratory-built prototype with a linear stage (a traveling range of 250 mm, Newport M-ILS250CCHA). A series of position-measurement experiments were performed with the position *δ*. It is noted that the linear stage can be moved directly via a linear motor featuring a closed-loop control scheme based upon a feedback signal generated with an optical encoder (resolution of 0.5 μm). Therefore, the final position was measured by using a position feedback signal from the optical encoder and its typical accuracy is ±1.7 µm. The experiments were conducted in a temperature-controlled laboratory. Here, the laser interferometer is replaced with a He-Ne laser (Newport R-30989) in the experimental setup for the measurement of the horizontal straightness errors, vertical straightness errors, pitch errors, yaw errors, and roll errors. 

**Figure 9:**
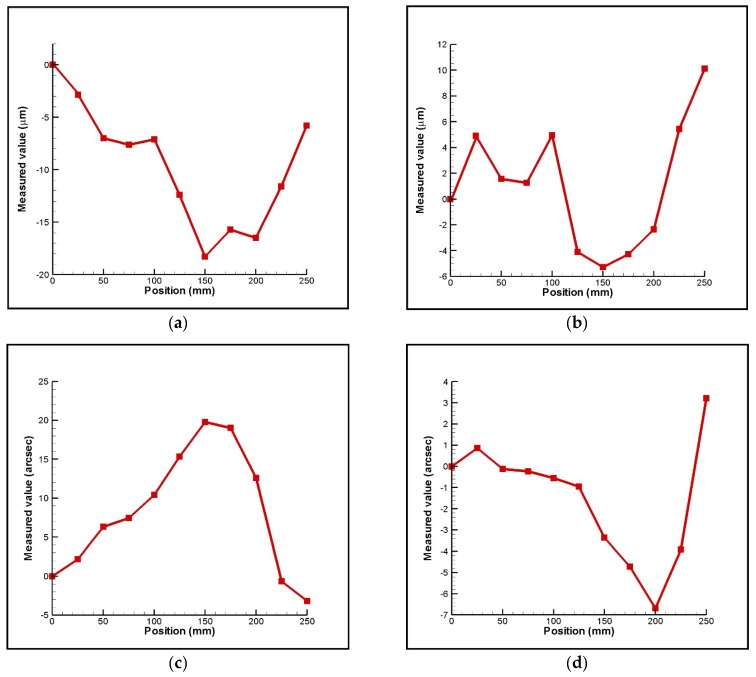

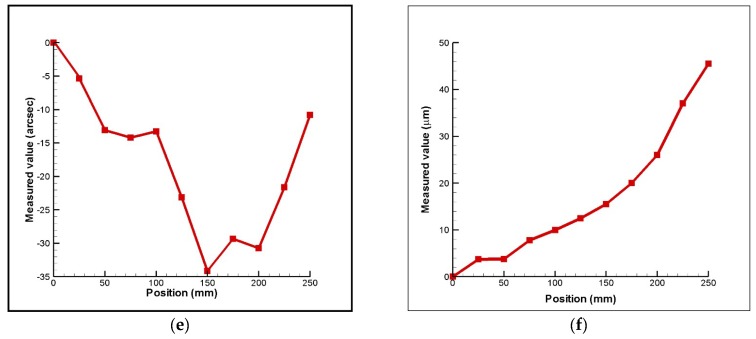
Experimental results for variation of geometric motion error with position: (**a**) horizontal straightness, (**b**) vertical straightness, (**c**) pitch, (**d**) yaw, (**e**) roll, and (**f**) positioning errors, respectively.

**Figure 10:**
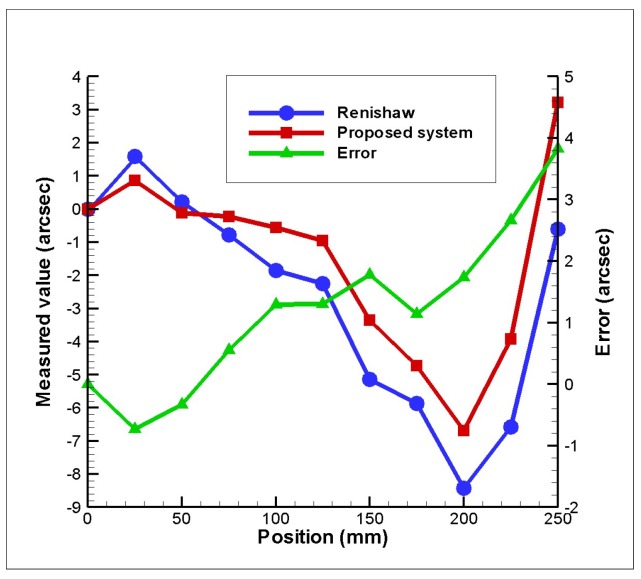
Comparative test results of proposed measurement system and Renishaw laser interferometer.

Figure 9 presents the experimental results obtained for 6DOF geometric motion errors of the linear stage with the imposed position, respectively. It can be seen in the resuslts that the horizontal straightness, vertical straightness, pitch, yaw, roll, and positioning errors of the linear stage are 19 µm, 16µm, 24 arcsec, 10 arcsec, 35 arcsec, and 45 µm respectively. It is noted that the measured positioning error increases when the imposed position increases and it dominates the geometric motion errors of the linear stage. Figure 10 shows the measurements of yaw errors on the linear stage using the proposed measurement system compared with measurements using the laser interferometer (Renishaw, Gloucestershire, United Kingdom, XL-80). It shows that measured accuracy of the proposed measurement system is about 4 µm when comparing to that of the laser interferometer. Consequently, it confirms that the proposed measurement system is suitable and has potential for simultaneous measurement of 6DOF geometric motion errors of a long linear stage. However, the measured accuracy of the proposed measurement system is not only influenced by the laser beam fluctuations but also by Abbe errors, misalignments, PSD sensitivity, aberrations, and so on [7,40]. Therefore, in order to improve measured accuracy, these issues must be considered and optimized in the future. 

## 5. Conclusions

This study has presented a new kind of non-contact optical measurement system for 6DOF geometric motion errors measurement of a linear stage of a machine tool. In the proposed approach, the commercial laser interferometer was combined into the proposed measurement system to measure the positioning error *δ_x_* for a long traveling range (> 200 mm). In contrast to conventional laser interferometers using only the interferometer method, the proposed measurement system can simultaneously measure six-degree-of-freedom geometric motion errors of a long linear stage with lower cost and faster operational time. The performance of the proposed measurement system has been evaluated using a laboratory-built prototype. The experimental results have shown that the proposed measurement system can simultaneously measure 6-DOF geometric motion errors of a linear stage for a long traveling range of 250 mm, and the measured accuracy of the proposed measurement system for yaw errors is about 4 µm when comparing to that of the laser interferometer.

## 6. Patents

The authors also published a Taiwan patent I614513 resulting from the work reported in this manuscript.
